# DOES PERCEIVED LONELINESS MATTER FOR DIVERSE OLDER MEN AND THEIR PROSTATE-SPECIFIC-ANTIGEN TESTING BEHAVIORS?

**DOI:** 10.1093/geroni/igz038.2384

**Published:** 2019-11-08

**Authors:** Tamara J Cadet, Shanna L Burke, Jamie Mitchel, Kyaien Conner, Frances Nedjat-Haiem

**Affiliations:** 1 Simmons University, Boston, Massachusetts, United States; 2 Florida International University, Miami, Florida, United States; 3 University of Michigan School of Social Work, Ann Arbor, Michigan, United States; 4 University of South Florida, Tampa, Florida, United States; 5 New Mexico State University School of Social Work, Las Cruces, New Mexico, United States

## Abstract

Evidence suggests loneliness is associated with poorer health practices and fewer health promoting behaviors, yet may be associated with greater use of the healthcare system. This dichotomy highlights the lack of understanding in the literature about the relationship between the experience of loneliness in early disease detection behaviors, such as prostate cancer screening and appropriate clinical interventions to support older men. Utilizing a series of logistic regression models, this investigation examined the relationship between loneliness and prostate cancer screening in 2008 and 2012 among White, Black, and non-Hispanic men, ages 50-74 years, (n= 4,875 for 2008 and 7.063 for 2012). The data source as the Health and Retirement Study. Findings indicate that White men were less likely to participate in PSA screening in 2008 if they felt left out or isolated. There was a reduced likelihood of screening among Black men who feel as though they have a lot in common with those around them in 2012. Utilizing approaches such as cognitive-behavioral therapy, motivational interviewing, and solution-focused practice, clinical social workers can have shared decision-making conversations to understand this phenomenon. Clinical social workers have unique training in the person-in-environment model that emphasizes the biological, psychological, social, and spiritual factors that influence behaviors such as cancer screening participation, that can help to understand men’s experiences, feelings or needs related to cancer screening participation. Given the lack of focus on men’s, this study provides formative data to test interventions to increase the well-being of older men.

